# Adipogenesis: A Necessary but Harmful Strategy

**DOI:** 10.3390/ijms20153657

**Published:** 2019-07-26

**Authors:** Mohammed El Hafidi, Mabel Buelna-Chontal, Fausto Sánchez-Muñoz, Roxana Carbó

**Affiliations:** 1 Departamento de Biomedicina Cardiovascular, Instituto Nacional de Cardiología “Ignacio Chávez”, México City 14080, Mexico; 2 Departamento de Inmunología, Instituto Nacional de Cardiología “Ignacio Chávez”, México City 14080, Mexico

**Keywords:** white adipose tissue (WAT), brown adipose tissue (BAT), angiogenesis, inflammation, hypoxia, lipolysis, lipogenesis, adipose tissue, circadian clock, Krüppel-like factors (KLFs), micro ribonucleic acids (miRNAs)

## Abstract

Obesity is considered to significantly increase the risk of the development of a vast range of metabolic diseases. However, adipogenesis is a complex physiological process, necessary to sequester lipids effectively to avoid lipotoxicity in other tissues, like the liver, heart, muscle, essential for maintaining metabolic homeostasis and has a crucial role as a component of the innate immune system, far beyond than only being an inert mass of energy storage. In pathophysiological conditions, adipogenesis promotes a pro-inflammatory state, angiogenesis and the release of adipokines, which become dangerous to health. It results in a hypoxic state, causing oxidative stress and the synthesis and release of harmful free fatty acids. In this review, we try to explain the mechanisms occurring at the breaking point, at which adipogenesis leads to an uncontrolled lipotoxicity. This review highlights the types of adipose tissue and their functions, their way of storing lipids until a critical point, which is associated with hypoxia, inflammation, insulin resistance as well as lipodystrophy and adipogenesis modulation by Krüppel-like factors and miRNAs.

## 1. Introduction

The energy metabolism of animal species is asymmetric and has the same behavior in their energy accumulation, which is necessary for survival during food shortage periods. Humans have a genetic programming that allows them to survive through famines. Thus, when there is enough food, fat accumulation supports survival during food shortage periods. Fat accumulation distribution has its functions, for example, the lower human body fat in females is much less metabolically active, and it is programmed to be metabolized during pregnancy and lactation. Meanwhile, abdominal fat depots engender a significant risk of metabolic imbalances [1].

In the prehistoric era, hunter-gatherers did not have what are called civilization diseases. These diseases are due to the low concentration of nutrients and their slower food transit through the digestive tract. Animal fat consumption was necessary for brain development. Furthermore, the domestication of fire made their diet more diverse, and they incorporated meat into their diet, thus allowing them the transition from a diet that secures only 20%–25% of the energy provided by plants to a high-energy diet that can help them to produce more muscle in order to hunt more efficiently [2]. As a consequence, the fat content in our diet was raised, and obesity became more frequent. As time passed, the human diet became poorer, and malnutrition rose, so obesity was considered a sign of wealth. However, recently, obesity has stopped being a sign of wealth and has become a sign of disease [3]. Hence the importance of adipose tissue and the need to understand it.

## 2. Adipose Tissue

Adipose tissue (AT) is considered to be the most variable and complex organ, regulating whole-body energy rather than that of one tissue. It is mainly composed of preadipocytes, vascular endothelial cells, infiltrating blood cells, pericytes, stromal, and regulatory T cells (T_reg_) [4], which were found to be involved in controlling the inflammatory state of the AT [5]. All these cells are in continuous interaction to tune the tissue expansion and the metabolic response.

The primary function of AT is to sequester lipids effectively to prevent lipotoxicity in other tissues, like the liver, heart, and muscle. Lipotoxicity affects membrane fluidity and alters membrane transport and, receptor signaling associated with the release of lipidic mediators and oxygen metabolites, which contribute to insulin resistance, inflammation, and cell death [6]. AT is continuously exposed to a slight remodeling. With positive energy, equilibrium changes occur as new adipocytes (hyperplasia), immune cells infiltration, angiogenesis and the enlargement of existing adipocytes (hypertrophy). All of these are considered processes associated with physiological AT expansion and are deemed necessary metabolic adjustments for adequate energy storage [7]. This storage is of lipids, because they have a high caloric value. The same amount of lipid contains double the energy (1 g = 38 Kj) of amino acids or glucose. Triglycerides (TG) are hydrophobic and insoluble in water, and this hydrophobic characteristic allows the cell to store many of them as lipid droplets [8]. In fasting conditions, it supplies energy by lipolysis [9].

During the development of obesity, AT suffers from inadequate remodeling and expansion, which frequently results in hypertrophy. Beyond a specific limit of AT expansibility and this depends on the size and number of adipocytes, as well as their functional properties [10]. The fat deposition, especially in the visceral compartment, is associated with metabolic risk factors and atherosclerosis, because free fatty acids (FFA) that can no longer be stored in AT constitutes ectopic deposits of lipids in other tissues in the body, promoting insulin resistance and metabolic syndrome (MetS) [11]. A mechanism that involves white adipose tissue (WAT) in the development of MetS and insulin resistance is its behavior as an endocrine organ, with a great capacity to secrete a wide range of molecules of various and multiple actions and contribute to several metabolic alterations, such as insulin resistance, the regulation of food intake and the inflammatory state [12]. The vasculature is essential for the healthy status of the AT. When there is deficient angiogenesis, the AT blocks its storage, a function resulting in slight hypoxia, which produces cytokines from all the cells of this complex organ [13].

Another tissue´s function is to mechanically cushion anatomical regions, such as the palms, buttocks, and heels, and internal organs, like the heart, adrenal glands, kidneys and ovaries [14]. There are different types of AT, which are broadly classified as WAT, brown adipose tissue (BAT) and bone marrow adipose tissue (BMAT). In contrast, there is another type of adipose tissue that plays a role in the transition between the WAT and the BAT, and it is called beige adipose tissue, which has unique characteristics.

### 2.1. WAT

WAT is regarded as a preferential organ, storing energy in the form of TG and cholesterol esters, controlling metabolism and secreting cytokines, proteins, lipids, and miRNAs [15]. The proteins secreted by the AT are termed adipokines. The most well-known of these is leptin, which provides satiety, augments lipid oxidation, and increases mitochondria biogenesis [16,17]. Adiponectin has anti-obesity, anti-diabetic and anti-inflammatory effects. Resistin has obesity effects, meanwhile Retinol-binding protein 4 has anti-obesity properties. Nefastin modulates appetite [18], Omentin improves insulin action and reduces obesity [19]. ApoM is a monitoring adipokine that modulates appetite [20]. WAT also has lipokines, such as Clb:17-palmitoate, fatty acid esters of hydroxyl fatty acids (FAHFA´s), monobutyrin and ceramides, which are lipids and are important for insulin sensitivity and decreasing the fat accumulation in this kind of adipocytes. WAT is classified as visceral (or upper) (VAT) and subcutaneous (or lower) (SAT) adiposity [18] (Table 1).

VAT includes the tissue around the heart and intraabdominal organs and is subdivided according to its corresponding localization: retro-peritoneal (rWAT), omental (oWAT), perigonadal (pgWAT), perirenal (prWAT), epicardial/pericardial and mesenteric (mWAT). It is a tissue that is very susceptible to apoptosis and has a lower level of lipoprotein lipase, high content of catecholamine-induced lipolysis and a high number of β-adrenergic receptors [25]. VAT is distinguished from other deposits by its direct connection to the liver via the portal vein, and it is positively correlated with glucose intolerance, plasma lipoprotein level alteration, elevated triglyceride, cholesterol concentrations and hypertension [26]. A decrease in VAT improves insulin sensitivity and glucose metabolism [27]. The analysis of insulin signaling pathways in human viscera and SAT shows that VAT expresses higher levels of proteins specific to the insulin signaling pathway and a higher sensitivity [28]. In summary, VAT is more sensitive to weight loss, more metabolically active, more lipolytic and produces more adipokines than SAT [29,30].

In humans during weight gain, AT extends not only to the current locations, such as SAT or VAT, but also to epicardium and perivascular space, which increases the risk of developing cardiovascular diseases. Recently, epicardial AT has been shown to have a significant secretory capacity of proinflammatory cytokines, such as interleukin-6 (IL-6), interleukin-8 (IL-8), and monocyte chemoattractant protein-1 (MCP-1), in response to treatment with vasodilator-stimulated phosphoprotein, which is involved in resistin-related endothelial dysfunction [31]. In patients with type II diabetes and MetS, epicardial adipose tissue is associated with reduced diastolic and systolic function and with both insulin resistance and left ventricular mass, with subclinical myocardial dysfunction, suggesting the inflammatory activity of epicardial AT-induced myocardial remodeling and dysfunction in middle-aged subjects with MetS [32,33].

Moreover, there is a direct relationship between the epicardial AT thickness and its inflammatory status and secretory profile, both modulated by statin therapy [34]. The epicardial adipocytes´ size is also associated with macrophage infiltration/polarization and the expression of toll-like receptors (TLR2 and 4) in coronary artery disease. Thus, the expansion of epicardial AT due to obesity may result in an elevated basal lipolytic rate, leading to an enhanced release of FFA, which activates TLR2 and TLR4 and modulates the nuclear factor κB (NF-κB) signaling pathway. This may, in turn, play an essential role in atherosclerosis development [35].

Concerning the perivascular AT, found surrounding arteries, recent studies have suggested a bidirectional paracrine and vasocrine-signaling pathway between the vascular wall and its perivascular AT [36]. In health, perivascular AT exerts a vasodilatation effect by the continuous release of perivascular AT-derived relaxing factors that enhance vasorelaxation via both endothelium-dependent and independent mechanisms and by the release of antioxidant molecules to avoid the reduced bio-availability of nitric oxide [37,38]. Besides, the differential abundance of brown adipocytes and white adipocytes in thoracic and perivascular AT, respectively, results in both an anti- and pro-atherosclerosis processes [39]. Because, under physiological conditions, WAT may act as FFA depots to prevent a rise in the circulation, BAT oxidizes large amounts of FA via thermogenesis [40]. Indeed, the cold exposure of apoE^−/−^ mice induces the activation of thermogenesis in perivascular AT and attenuates the atherosclerotic process, whereas in mice without perivascular AT, such protection is lost [41]. In metabolic diseases, such as obesity, a shift in the adipocyte phenotype from a protective profile to an imbalanced production of proinflammatory, pro-oxidant and profibrotic adipokines, such as leptin, resistin and visfatin, has a direct local effect in the pathogenesis of arteriosclerosis via an increased rigidity of the wall, contributing to the hypertension found in the obese patient [42].

SAT is molecular and phenotypically different from VAT. It improves metabolic parameters and insulin signaling, so SAT accumulation may prevent MetS. This tissue is subdivided into gluteal, femoral, and abdominal, which is considered superficial, and humans also have deep SAT (dSAT), which has an increased expression of inflammatory cytokines and saturated FA and may be considered to be as risky as VAT [43]. It appears that obesity is associated with a preferential increase in the deep layer, and weight loss in obese subjects preferentially affects this layer, suggesting that the deep subcutaneous layer is more active than the superficial layer [44].

Dermal (dWAT) is a type of SAT that has the function of healing, covering the hair follicles and thermogenesis. This tissue is associated with dermal skin layers [45].

Another type of SAT is the pink one (piSAT). This kind of SAT comes from a population of adipocytes from the mammary glands, and it is considered to be an example of strike plasticity. During lactation and pregnancy, these adipocytes transdifferentiate to milk-producing cells and return to adipocytes after the post-lactation period. In the post-lactation period, these multilocular adipocytes have milk protein granules. Cinti et al. found that, during mammary gland involution, some secreting epithelial cells in the subcutaneous fat depots may transdifferentiate to BAT [24].

Preadipocytes are very important for the AT, since the characterization of their lineage in each depot has contributed to AT biology. WAT has three distinct types of preadipocytes, characterized by their unique high expression of marker genes. These are Wilms tumor 1, transgelin and myxovirus 1. They respond differently to exogenous stimuli, and all three correspond to an independent WAT subpopulation [14]. These cells also have numerous markers, such as preadipocyte factor 1 (Pref-1) and platelet-derived growth factor beta (Pdgfr), which are present in adipogenicity [46,47].

Rodeheffer et al. showed that preadipocytes lack endothelial cell (CD31), macrophage (MФ) (CD45) and erythrocyte (Ter119) markers and possess stem cell markers, such as CD29, CD34, Sca1 and CD24, which are adipogenic subpopulations [48]. Preadipocytes from a single fat depot have many developmental origins [15]. Each population differs in terms of the beiging capacity, differentiation, wound healing, vasculogenesis and remodeling properties.

Schwalie et al. discovered that there is a new adipocyte subpopulation, CD142+, termed adipocyte regulator cells, which have paracrine actions in regulating adipocytes formation and are more abundant in VAT than in SAT and increased in obesity [49]. Another subpopulation is the fibroblast-specific protein-1, which is necessary for maintaining the preadipocytes pool and its adipogenic potential [50].

The lineage of preadipocytes is heterogenous, pgWAT, in which it has been shown that there are adipocytes derived from multiple lineages, is an example of this. pgWAT from males are from both the paraxial and lateral plate mesoderm, while in females, they are only from the lateral plate mesoderm [15]. Another example is prWAT, which has a high percentage of rapidly dividing preadipocytes, indicating its possible embryonic origin [51].

#### 2.1.1. WAT and the Regulation of Energy Homeostasis

Lipid metabolism in AT is regulated at three levels: the uptake of FFA, lipogenesis, and lipolysis. Each of these processes is controlled by external stimuli, including insulin, catecholamines, and cytokines.

The excess energy storage, in the form of FAs, is one of the primary functions of the adipose tissue. The size of the adipocytes can vary greatly, up to 20 times, in storing FFA in the form of TG in the lipid droplet (lipogenesis). Conversely, during periods of dietary restriction and in the case of energetic need, TGs are hydrolyzed (lipolysis) to FFA and released into circulation. Then, mobilize to other tissues in response to metabolic needs [52].

#### 2.1.2. Lipogenesis

The synthesis of TG in adipose tissue may be considered a detoxification process of the excess FFA to avoid lipotoxicity and metabolic disease complications, such as insulin resistance, MetS, type II diabetes, and atherosclerosis.

The TG stored in the adipocytes is synthesized from FA and glycerol, both of which must be activated to become FA acyl-CoA and glycerol-3-phosphate, respectively. This is required for the initial step of the TG synthesis, which is produced either from glucose by glycerol kinase activity in the early stages of glycolysis or from gluconeogenic precursors by glyceroneogenesis. Glucose enters the adipocytes using glucose transporters 1 and 4 (Glut1 and Glut4), responsible for basal- and insulin-stimulated glucose entries, respectively [53].

Regarding TG synthesis in adipocytes, FA is mostly used for this synthesis from circulating lipids. The exact site of TG synthesis, and how new TGs are directed to lipid droplets, is still debated. The circulating FAs are lipoprotein-incorporated TGs or albumin-bound non-esterified FAs. The lipoproteins-incorporated TGs must first be hydrolyzed out of adipocytes by lipoprotein lipase (LPL), an enzyme linked to AT capillaries [54]. In transgenic mice with an over-expression of LPL, the increased adipose tissue lipid storage is associated with glucose and insulin tolerance, as well as an increased energy expenditure in the mice, when they have a high-fat diet [55]. Furthermore, the increased adipose tissue LPL is associated with adipocyte hypertrophy in SAT, reducing visceral adiposity and metabolic risk in obese, older women, a process giving SAT a protective role [56]. However, a lower adipose tissue LPL activity and a limited capacity for subcutaneous adipocyte expansion are associated with MetS development in postmenopausal women with visceral obesity [57].

Concerning the albumin-bound non-esterified FAs, their capture by adipocytes requires specific processes to allow them to cross the plasma membrane. Human white adipocytes express different FA transporters, which facilitate and control their transport: The 36-protein Cluster of Differentiation (a homologue of the murine fatty acid translocase); the FA transport protein; and the fatty-acid-binding protein (FABP). The human white adipocytes express two FABPs: adipocyte Protein 2 (aP2) and FABP4; this last is secreted from adipocytes and acts as an adipokine for the development of insulin resistance and atherosclerosis [58,59]. Thus, WAT may appear as the protector in the context of its capacity for unlimited lipid accumulation. However, there are several causes and consequences of different metabolic phenotypes, such as the insulin-sensitive phenotype, that make AT lose its plasticity and flexibility in the metabolic process and thereby its role as a reservoir or buffer against FFA fluxes.

##### De Novo Lipogenesis

De novo lipogenesis (DNL), is the synthesis of FAs from glucose including several metabolic steps such as glucose transport, cytoplasmic glycolysis, mitochondria citrate release. Enzymes involved in these pathways include glucose transporters (GLUT), glucokinase (GK), lipogenesis enzymes, such as acetyl-CoA carboxylase (ACC), fatty acid synthase (FAS), and stearoyl- CoA desaturase 1 (SCD-1). These enzymes catalyze the synthesis of monounsaturated fatty acids and glyceraldehyde 3-phosphate acyltransferase (GPAT) allowing the synthesis of TG [60]. The majority of these enzymes are controlled in the short term by post-translational and allosteric mechanisms. However, the main regulation is long-term transcription, which is tightly controlled by nutritional conditions as the carbohydrate rich diet that increases blood glucose and stimulates insulin secretion. Insulin in turn stimulates the expression of the transcriptional factor sterol regulatory element binding protein (SREBP) and carbohydrate responsive element binding protein (ChREBP), major players in DNL in adipose tissue and other tissues such as liver and muscle [61]. Under physiological condition, hepatocyte and adipocyte DNL are differentially regulated at the transcription level and are synergistically regulated by signals from the peripheral tissues. However, under pathophysiological as obesity, ChREBP, that mainly controls DNL in adipocytes, is increased in the liver from obese compared to lean subjects, whereas the expression decreased in adipose tissues [62]. This disruption of the balance between adipocyte and hepatocyte DNL, leads to increased DNL in the liver and contributes to fatty liver and other relevant metabolic diseases [63]. Besides, the reduction of DNL by the liver-specific deletion of SREBP cleavage activating protein (SCAP), a protein required for cleavage of SREBP1c, is accompanied by increased adipose tissue DNL associated with improved fasting glycemia, glucose tolerance and insulin sensitivity [64]. In addition, genetically deleting adipose tissue lipid chaperones aP2 and mal1, adipose DNL increases and renders mice resistant to diet-induced obesity, fatty liver disease, insulin resistance and Type 2 diabetes [65]. These finding support the view that adipose DNL, unlike hepatic DNL, may be metabolically beneficial.

#### 2.1.3. Lipolysis

In adipocytes, the lipolysis of TGs into FFA and in glycerol occurs when energy is needed during fasting or intense exercise training. FFAs released into circulation serve as an energy source for metabolically active tissues, such as the muscles, heart, and brain, where their oxidation generates ATP.

To generate FFAs in adipocyte, TGs are successively hydrolyzed to diacylglycerols (DAG) by adipose triglyceride lipase (ATGL), and then the DAG is hydrolyzed to monoacylglycerols by hormone-sensitive lipase (HSL). Ultimately, three molecules of FA and one molecule of glycerol are released from the adipocyte. In adipocytes, lipolysis is regulated by hormones or mediators (adrenaline, norepinephrine and glucagon) that activate the adenylate cyclase system, leading to an increased intracellular concentration of cyclic AMP. This activates protein kinase A (PKA) phosphorylation, which, in turn, activates the HSL by phosphorylation [66]. The HSL activation is part of the metabolic adaptations to the negative energy balance, typical of the mammalian [67]. Conversely, the inhibition of the expression of HSL in mice results in the accumulation of DAG, a physiological activator of protein kinase C (PKC), leading to insulin resistance in AT [68]. DAG is also a substrate of diacylglycerol kinase, and its deficiency results in abnormal lipid metabolism, such as obesity and insulin resistance [69]. Several other factors have been found to induce lipolysis. Cytokines increased the phosphorylation of HSL, which was reversed by treatment with DPI and of NADPH oxidase 3 (NOX3) expression, silently suggesting the participation of NOX3-mediated superoxide production in the increased adipocyte lipolysis in inflammatory settings [70]. Leptin also acts directly to induce lipolysis in bovine adipocytes by increasing the translocation of ATGL and HSL to lipid droplets [71]. Thus, adipocytes can accumulate a considerable amount of FA under TG and cholesterol ester forms, and these are stored in intracellular lipid droplets, which are surrounded by proteins called perilipins or Plins.

#### 2.1.4. Lipid Droplets in WAT

Lipid droplets are intracellular structures that store neutral lipids. For a long time, they were considered as simple inert energy storage sites, and their volume may increase or decrease according to metabolic energy needs. However, they have recently attracted considerable interest and are now considered as dynamic structures, permitting lipid exchanges between different intracellular compartments, such as endoplasmic reticulum, mitochondria, endosomes, peroxisomes or plasma membrane, particularly by providing them with lipid for their membrane expansion [72]. In adipocytes, lipid droplets are particularly well-developed organelles and exhibit unique properties to store lipid in excess and release it when metabolic energy is needed.

In contrast to vesicular organelles, which are delimited by a phospholipid bilayer, lipid droplets are surrounded by only a monolayer of phospholipids, which delimits the very hydrophobic content of TG and cholesterol esters [73]. Different proteins are localized on the surface of lipid droplets. The best known are perilipins or Plins, which stabilizes these organelles and regulates lipolysis in AT.

In mammals, five separate single-copy genes of perilipin are identified as *Plin1*, *2*, *3*, *4*, and *5* [74]. The expressions of several lipid droplet-associated proteins, including Plins, in mammals are regulated by the peroxisome proliferator-activated receptor (PPAR), a family of transcription factors that are activated by long-chain fatty acid ligands [75,76].

Plin1 is expressed in both WAT and BAT and serves as a physical barrier by a selective recruitment site for intracytoplasmic lipases to protect the lipid droplet content [77]. This protective role of Plin1 is evidenced by the phenotype of mice, whose Plin1 gene was deleted. Indeed, these animals are lean and show a limited lipid storage due to excessive basal lipolysis [78]. The phosphorylation of Plin1 regulates its role in the lipolysis activity by PKA, which is activated by the increased cyclic AMP during fasting, a physiological situation of increased AT lipolysis releasing FA [79]. Thus, it seems that the phosphorylation of Plin1 promotes the anchoring of lipases on the surface droplets. Besides, the Plin1 overexpression was found to up-regulate the expression of SREBP-1c, and its target gene, diacylglycerol acyltransferase [80]. The over-expression of Plin1 also inhibited the expression of lipolysis enzymes of HSL and the expression and content of TNF-α, IL-1β, and IL-6, induced by the lipopolysaccharide in cow adipocytes [81]. In mice, Plin1 deficiency impairs peripheral insulin sensitivity, accompanied by a reduced fat mass and the promotion of the secretion of pro-inflammatory lipid metabolites, such as prostaglandins, which potentiated monocyte migration [82]. Plin2 and Plin3 are widely distributed in the majority of cell types. Recently, it was found that reactive oxygen species (ROS) up-regulate Plin2 expression in hepatocytes, promoting lipid droplets and resulting in lipid accumulation in liver tissues [83]. Moreover, the ablation of Plin3 in mice enhanced basal and stimulated lipolysis in inguinal WAT, inducing PPARα activation [84].

Besides Plin4, Plin5 is the most extensively studied lipid droplets surface proteins in terms of its direct role in managing lipid in the heart and skeletal muscle. The expression of Plin5 in the heart by an elevated FFA supply from AT by fasting increased the cardiac TG levels and lipid droplet size, suggesting that the interface between lipid droplets and cardiac mitochondria represents an organized and dynamic “metabolic synapse,” which is highly responsive to FA trafficking [85]. Plin5 expression in skeletal muscle also plays a vital role in the regulation of lipid droplet metabolism and the control of intracellular FA fluxes. The increased Plin5 expression in hepatocyte or in skeletal muscle, which occurs with overnutrition may play an essential role in preventing hepatic insulin resistance [75,76]. On the other hand, Lipin5 deletion in hepatocytes from Plin5LKO mice exhibited reduced contact between lipid droplets and mitochondria, which decreased FA oxidation and reduced TG secretion and hepatic insulin resistance [86].

The lipid droplet expansion in adipocyte may provide a means of lipotoxicity protection in response to FFA overload. Perilipin proteins mediate this protection by their overexpression and activity, regulated by phosphorylation, to modulate the recruitment of different cytoplasmic lipases in order to release FFA, which accumulates in ectopic deposits and contributes to the development insulin resistance, type II diabetes and atherosclerosis [87]. Targeting these proteins surrounding lipid droplets may help to control lipolysis, but not the expansion of adipocytes. Indeed, adipocyte hypertrophy in SAT due to increased LPL expression activity is associated with reduced visceral adiposity and a metabolic risk in obese patients, giving SAT a protective role [88].

#### 2.1.5. The Circadian Clock in Adipogenesis

Cellular clocks generate the rhythmicity of most metabolic functions, and the minimal unit for autonomous circadian rhythmicity is a single cell. There is also evidence that proteins, lipids and organelles function according to circadian rhythmicity.

The circadian clock (CC) characterizes the 24 h cycle oscillation, which serves to regulate the significant physiological activities. It regulates the sleep/wake cycle, endocrine and rhythms, and fast/feeding cycles. The CC desynchrony contributes to increasing morbidity [89].

This system is located in the suprachiasmatic nucleus of the hypothalamus, and there are clock genes that coordinate the CC function and modulate the lipid metabolism and also a relationship between hypoxia and CC. Many genes are involved in the CC, and the response to hypoxia is deregulated under disease and stress conditions [90]. Some reports also mention that molecular clockwork affects the expression of VEGF during hypoxia [91].

The primary genes are BMAL1, CLOCK, ARNT3, Period (PER1, PER2, PER3), crytochrome (CRY1, CRY2) and MOP3. This clock is composed of activators (CLOCK/BMAL1) and repressors (PER/CRY), and they interact, repeating themselves every 24 h [92].

BMAL1 is very important in the modulation of lipid synthesis and fat storage, utilization and adipocyte differentiation. BMAL1/ CLOCK cooperates with SREBP-1c, downstream genes, like FAS, 3-hydroxy-3- methylglutaryl-CoA reductase (HMGCR), fatty acid elongase family members (ELOVL), the low-density lipoprotein receptor (LDLr) and acetoacetyl-CoA synthetase (AACS), to modulate liver lipid metabolism [93]. BMAL1 also functions as a cAMP-responsive coactivator of HDAC5 in hepatic gluconeogenesis [94]. BMAL1 can initiate PPARs transcription, while PPARs can turn on BMAL1 transcription, driving PPARα expression. BMAL1 regulates the 24-h expression of KLF10. This transcription factor can modulate the circadian gene expression in lipogenesis, gluconeogenesis, and glycolysis and with reciprocal control between PPARα/Bmal1, PPARγ/Bmal1 and KLF10/Bmal1, providing excellent metabolic regulation in the circadian rhythms system [95]. Similarly, CLOCK is closely associated with NAFLD and is mainly expressed in the peripheral tissues [96,97]. Any alteration in the homeostasis of the circadian cycle is known to cause abnormal storage by adipocytes.

### 2.2. BAT

BAT is a constitutive and thermogenic tissue, with smaller adipocytes and a high mitochondria density [15], and it is specialized for heat generation, rather than producing ATP, with high levels of uncoupling protein 1 (UCP1) [98,99,100,101]. AT is innervated by the sympathetic nervous system, which plays the role of activating thermogenesis releasing noradrenaline through its specific receptors. β-adrenergic signaling in BAT activates peroxisome proliferated receptor γ coactivator 1 α (PGC-1α), stimulating UCP1. Cold is another UCP1 inducer, stimulating the lipid uptake for efficient heat production and mitochondrial biogenesis [102] and also induces the expression of pro-angiogenic factors, such as a vascular endothelial growth factor (VEGF), indicating that the angiogenic remodeling of BAT is necessary for thermogenesis. Sánchez-Gurmaches and collaborators, provide evidence that suggests that AKT2 kinase drives de-novo lipogenesis by stimulating ChREBPb protein activity in adipocytes, and cold induces the AKT2-ChREBP pathway to optimize storage and thermogenesis in BAT [103]. This tissue presents an increased expression of PPARγ and PGC-1α, and is preferably located in the supraclavicular and paravertebral zones [26] where it has its own identity and a different origin from WAT, because brown fat precursors express a myogenic gene signature, as WAT needs the bone morphogenetic protein 2 and 4 (BMP2 and BMP4) to differentiate; as well as BMP7 and fibroblast growth factor (FGF) [104]. This tissue also requires estrogen-related receptor gamma (ERRγ) to maintain its BAT identity [105], and is susceptible of chronic inflammation. Like WAT, this kind of tissue can suffer from alterations by the deficiency of IL-10, which is an anti-inflammatory cytokine [106]. BAT is present in animals that hibernate and have to acclimate to cold seasons and also in human adults as well as in infants [99].

AT has a very high plasticity capacity and does not remodel only to perform its physiological role, but also to change its characteristics and transform itself into another type of tissue, so that not all brown-like fat cells come from precursors expressing the myogenic lineage, and these non-classic brown adipocytes are called beige cells, [107] and beiging is a remodeling process by the repeated trans-differentiation of mature WAT into beige adipocytes.

### 2.3. Beige Adipose Tissue

**Figure 1:**
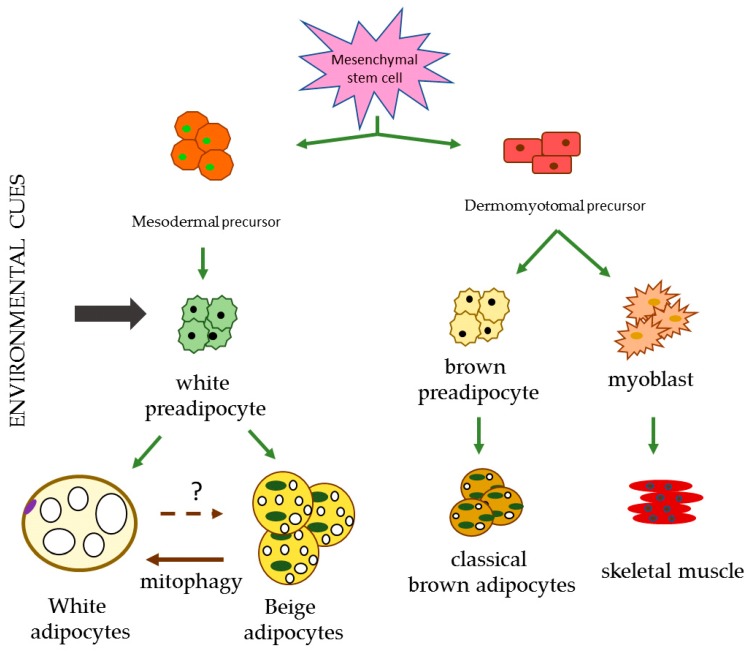
Different lineages responsible for the differentiation of the diverse types of adipocytes. Environment cues include cold, exercise, ligands and cachexia. Created from Towsend et al. and Ikeda et al. [104,107].

Beige AT is a transient distinct type of adipocytes found in WAT depots as well as in BAT. It is considered an inducible form of thermogenic adipocytes that sporadically reside in the WAT. Like BAT, they possess abundant cristae-dense mitochondria and express the UCP1 protein [108]. They differ from the classic adipocytes due to their ability to function as a lipid storage or to produce heat [109]. Given its thermogenic ability, they have a high UCP1 content. These adipocytes share some features with WAT as a mesodermal progenitor, and with BAT, as dermomyotomal [104], but they are unique for their distinct gene expression profile and adult origin [109] (Figure 1). The adipokines present in this tissue are FGF21s, which activates PGC-1α, BMP4, and BMP2 [18]. Additionally, Irisin is an adipokine that is newly related to obesity and present in the beige tissue. Eventually, this protein was related to exercise, but now it has been observed that preadipocytes possess the receptor of this protein and that they are present in the adipocytes that go through the browning process. This phenomenon is related to fibronectin and α-integrins [110].

Wolfrum et al. put forward the hypothesis that beige tissue arises from WAT by a direct process, and beige and WAT interconvert bidirectionally, depending on the stimuli, such as cold or β-adrenergic signals, activated by the sympathetic nervous system [111]. Beiging recruitment is very versatile, and is promoted by exercise, cold [112,113]; disease or trauma [114,115]; hormones and cytokines [116,117,118,119,120,121], drugs [122,123]; calorie restriction and nutritional modifications [124,125,126] and signaling molecules [127,128,129,130].

### 2.4. BMAT

Bone and fat arise from the same mesenchymal progenitor. Bone marrow adiposity begins in the early stages of life (before or at birth) and proceeds to grow, replacing hematopoietic tissue. It has been reported that a mixed population of BAT/WAT adipocytes are present. BMAT is composed of adipocytes that contain lipids and are identified by a positive stain for perilipin [131]. Lipolysis from BMAT provides osteoblasts with FFA to produce bone turnover [132].

BMAT occupies 50% to 70% of total bone marrow volume and its evolution proceeds from a red to a yellow conversion in a centripetal fashion, histologically resembling WAT [133].

This tissue does not reflect the excess of fat deposited outside, and it is considered a dynamic and responsive tissue, so it can react to nutritional, environmental and hormonal stimuli [131]. BMAT secretes adipokines and is a significant source of adiponectin [132].

BMAT is subdivided into a constitutive type (cBMAT), which is present from birth and concentrated in the distal skeleton and, has larger adipocytes and a non-hematopoietic activity and, regulated type (rBMAT), which is found in the more hematopoietic active sites, like the spine and proximal limbs, which also respond to exogenic factors. rBMAT develops after cBMAT [133] and is characterized by an elevated presence of beige adipose tissue markers, such as UCP1 [134].

In some conditions, such as aging, type II diabetes mellitus, anorexia nervosa and calorie restriction, this tissue is associated with an aberrant turnover, with marrow adipocytes infiltration and bone remodeling formed by an increased resorption and bone formation suppression [135].

## 3. Adipogenesis in Response to an Overflowing Storage Capacity

De novo adipogenesis is needed when adipocytes reach the upper volume limit, and new cells are therefore required to increase the storage capacity (hyperplasia). This kind of adipocytes activates genes and proteins (PPARγ, C/EBPα, SREBP1c, fatty acid synthase (FAS)) to promote adipogenesis [5].

When there is an unhealthy storage of lipids, adipocytes start to become hypertrophic to the point of no return. Hypertrophic adipocytes have a unilocular-like lipid droplet, and one of the first events during the high lipid storage is mitochondria dysfunction [136]. They also have a disorganized cortical actin and an impaired glucose transport translocation. They have an abnormal function, which leads to insulin resistance, necrotic behavior and dead cells in obesity, thus producing an inflammatory state. They are also characterized by local hypoxia, beginning with angiogenesis, apoptosis and the secretion of adipokines as well as miRNAs [18].

The most recent information indicates that the process that causes the imbalance within the adipose tissue is as follows: Lipids and adipose adipokines causes a low-grade inflammatory state and trigger mitochondrial dysfunction, leading to ROS production, cell death, inflammation and metabolic dysfunction [136]. These organelles are essential for maintaining the metabolic homeostasis in the AT and secreting of adiponectin [137].

Adiponectin is the most copious cytokine in the AT, has favorable effects on the atherosclerotic process and systemic inflammation, and has been found in lower levels in obese subjects [138]. Indeed, hypertrophic adipocytes lose their functional activities and the production of adiponectin [136].

**Figure 2:**
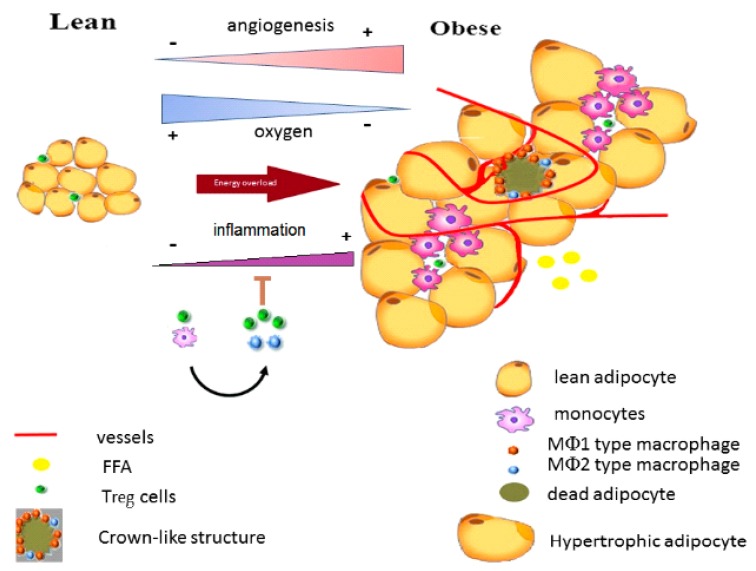
The cellular transition during energy overload. FFA: free fatty acids; Treg: T regulatory cells; and MФ: macrophages. Based on Khodabandehloo et al. [139].

Adiponectin decrease leads to the initiation of the inflammatory state through the infiltration of macrophages releasing nitric oxide, producing a pseudohypoxia, as well as producing fibrosis and cell death, and on the other, it inhibits the differentiation of new adipocytes. Then, the vascularization process begins due to an incipient hypoxia [136] (Figure 2).

### 3.1. Hypoxia

Previously, it was proposed that hypoxia was the cause of the dysfunction process. Due to the enlargement of the cells, their expansion provokes a lack of vasculature, and the hypoxic state begins to occur [140]. Now, the story is different, as mentioned above.

The partial pressure of oxygen in lean AT is about 48 mmHg, meanwhile, in the AT of an obese individual, it is 12 mmHg. Metabolic disturbances are evident, and one is the excess of lactate, a product of glycolysis, which also increases the number of Glut1 favoring the entrance of glucose, as a consequence of the demand. Besides, the enzymes of the glycolytic pathway, such as hexokinase 1 and 2 (HK1 and HK2), glucose-6-phosphate isomerase (GPI), phosphofructokinase (PFK) and aldolase C (ALDOC), are increased [141].

During hypoxia, besides the adiponectin concentrations reduction, mitochondrial respiration and biogenesis are lower, chemerin expression is enhanced, leptin concentrations rise, and UCP-2 is reduced. Thus, there is scarce communication and disorder, very poor orchestration and dysfunction [141].

On the other hand, hypoxia induces the expression of angiogenic factors, such as VEGF, an essential fibroblast growth factor and leptin. Additionally, there is an activation of inflammatory response-associated genes, such as hypoxia-inducible factor 1 alpha (HIF-1α). HIF-1α is a transcription factor that accelerates fibrosis, leading to necrosis and, also the augmentation of local inflammation. Coactivators regulate HIF-1α as p300 and CBP, which catalyze the acetylation of histone proteins and the initiation of the transcription. The corepressors activity is determined by histone deacetylases (HDACs). However, HIF-1α inhibition is due to the orchestration of HDAC3, thyroid hormone receptors (SMRT) and the nuclear corepressor (NCoR), which form a complex of proteins [142]. In this situation, there is a decrease of mesenchymal stem cells, which causes a reduction in the adipocyte differentiation and redirects this mesenchymal cell to a myogenic lineage.

Angiogenesis began as a solution to the lack of oxygen and attempt to provide nutrients to dying tissue. It has been reported that the capillary density is lower and more extensive in obese subjects than in lean subjects. As a consequence, MФ began to migrate to digest dead and dying adipocytes, joined to adipocyte apoptosis, and cell survival and fibrosis is not closely associated with this process. There are two types of MФ, depending on its functions in the AT. M1Ф, known as classically activated, express TNFα, IL-1β. and iNOS. These cells are the major contributors to inflammation and insulin resistance. They can be recruited by infection too.

Meanwhile, M2Ф express arginase and alternatively, IL-10 and Ym-1 and are called, activated. The arginase is present to sequester arginine from iNOS and lower the nitric oxide concentration and hence the suppression of M1Ф. IL-10 is an anti-inflammatory cytokine that alleviates the TNFα-induced insulin resistance [143].

Invariant natural killer T cells (iNKT) in the AT secrete IL-4 and -10, with unique functional characteristics to regulate T_reg_ cells by producing IL-2 and M2Ф via IL-10 production [144].

MФ in the AT release metalloproteinases (MMP) to promote endothelial cell-tube formation by MMP-9, which are part of the extracellular matrix remodeling during adipogenesis. MФ also remove lipid leakage from AT, transforming themselves into lipid-loaded MФ, resembling foam cells, causing more inflammation and forming plaque in the arteries. This increases the risk of obesity causing myocardial infarction (MI) [145].

Adipocytes death by hypoxia is directly related to a chronic low-grade inflammation in obese AT [143,146,147], which is thought to contribute to the development of the sequelae of obesity.

### 3.2. Inflammation

#### 3.2.1. Inflammation in Dysfunctional Adipocyte by Obesity

The increase of interest in the study of AT in recent years has been caused by the awareness of its essential role in maintaining metabolic homeostasis. It has the ability to rapidly expand or reduce in periods when food is either abundant or during fasting and energy expenditure. Moreover, there is a component of the innate immune system [148], far beyond being an inert mass of energy storage.

#### 3.2.2. Chronic Low-Grade Inflammation in Obese AT

Adipokines refer to a large number of secreted proteins (hormones and cytokines) from AT, of which the most studied are adiponectin and leptin [149]. Adipocytes have an inherent ability to secrete adipokines, both anti- and pro-inflammatory [150]. However, hypertrophic adipocytes produce an abnormal amount of adipokines and stimulate chemotaxis to recruit local or circulating MФ [149]. In obese AT, there is an increased production of TNF-α, IL-6, MCP-1, iNOS, TGF-β1, and procoagulant proteins, such as plasminogen activator inhibitor type 1, tissue factor and factor VII [151,152,153,154,155,156,157]. The action of cytokines, TNF-α and MCP-1, has the effect on adipocyte function of increasing lipolysis and decreasing triglyceride synthesis [149]. While during prolonged overnutrition, adipocytes cause an inflammatory response due to adipokine secretion, macrophages are responsible for most of the cytokine production in obese AT [158].

Macrophages of AT from lean mice are M2Ф, whereas the MФ of AT from obese mice are predominantly M1Ф [143]. M2Ф macrophages display anti-inflammatory properties and are associated with angiogenesis and wound repair, whereas M1Ф macrophages are related to inflammation [146]. In obesity, it has been found that a shift in the activation state of macrophages in AT from M2Ф in lean animals to M1Ф [143], upon the activation of MФ, secretes numerous cytokines and chemokines, such as TNF-α, IL-1, IL-6 and chemokine MCP-1 [159,160].

MФ, representing around 5% of the AT in a lean state, increases drastically in obesity [161,162]. This increase is due to the formation of the so-called “crown-like structures” (CLS). Produced by the infiltration of MФ, which phagocyte death adipocytes [163,164], this is a hallmark of the chronic low-grade inflammation in obese AT and is implicated in the metabolic complications of obesity. Exercise training has been associated with a reduction of adipocyte death and thus a decrease in chronic inflammation by inhibiting MФ infiltration in AT [165]. On the other hand, although chronic inflammation triggers pathophysiological sequelae due to obesity, pro-inflammatory signaling in the adipocyte is required for AT remodeling and expansion [166].

It is worth mentioning that AT inflammation is also produced during weight loss by short-term caloric restriction or a bariatric procedure and not only during fat accumulation and weight gain [167]. Therefore, extreme or fast changes in body fat can trigger an inflammatory response [168].

As MФ clean death adipocytes, they produce toxic products, such as ROS and reactive nitrogen species, that contribute to the damage response, surrounding AT, and promote fibrosis. Besides, they essentially induce an early removal of MФ for proper wound healing. However, in obesity, the damage persists, resulting in AT dysfunction [169]. Evidence shows that many comorbidities in obesity are associated with chronic inflammation, such as type II diabetes mellitus, steatohepatitis, non-alcoholic fatty liver, asthma, cardiovascular and neurodegenerative diseases [170]. A further advance in the understanding of the inflammatory response in obesity can lead us to the development of new approaches to treating the associated diseases.

#### 3.2.3. Oxidative Stress in Obese AT

The production of ROS by immune cells contributes to the oxidative state in obese AT, which is an instigator of MetS. In obese AT, there is both an increase of oxidative stress and reduced antioxidant defense, the latter of which is due to the action of inflammatory cytokines, which down-regulate glutathione S-transferase A4, peroxiredoxin 3 and glutathione peroxidase 4 [148,171]. In AT, the unsaturated FAs are targets of oxidation by ROS. Oxidized lipids and also oxidized proteins (protein carbonylation) accumulate in higher concentrations in visceral depots than in subcutaneous depots and have been implicated in the control of insulin signaling and, glucose and lipid metabolism [148]. Remarkably, it seems that oxidative stress precedes inflammatory cell infiltration into AT, since oxidative injury was detected in biopsies when inflammatory cell markers were absent [169,172,173].

NADPH oxidase 4 (NOX-4) is the major isotype expressed by adipocytes and is activated under overnutrition. It is also able to produce, not only superoxide, but also hydrogen peroxide [174]. The inflammatory cytokine, IL-1β, activates NOX-4 expression, increasing ROS production in adipocytes [97]. It has been proposed that the superoxide anion, produced by NOX, is involved in insulin resistance in the liver [54]. Indeed, overproduced superoxide in an obesity condition [175] is a significant contributor to insulin resistance in obese AT [148].

#### 3.2.4. Insulin Resistance

One of the chronic low-grade inflammation consequences in AT is insulin resistance. TNF-α, produced mainly by the MФ of AT, was the first cytokine demonstrated to directly impede insulin action in the adipocytes [151]. It down-regulates the primary insulin-responsive Glut4 and inhibits the insulin-dependent tyrosine phosphorylation of the insulin receptor and insulin receptor substrate-1 through the stimulation of sphingomyelinase activity [143,176].

Leptin, which is augmented in obesity, also up-regulates pro-inflammatory cytokines, such as TNF-α and IL-6, which are associated with insulin resistance and the development of type II diabetes [177]. Besides, TNF-α and IL-6 are associated with the down-regulation of adiponectin [147,178,179]. Adiponectin is a hormone that increases FFA oxidation in several tissues but which also enhances insulin sensitivity in the muscle and liver. Thus, reduced adiponectin concentrations are correlated with insulin resistance and hyperinsulinemia [180]. Moreover, IL-6, which is highly expressed in VAT, is directly related to the cause of liver insulin resistance, and an increase in the IL-6 concentration is a predictor of the development of type II diabetes [181].

## 4. Lipodystrophy (LPD)

Lipodystrophies are a heterogeneous group of diseases, represented by the selective absence of adipose tissue or the loss of functional adipocytes, which leads to dyslipidemia, ectopic steatosis and insulin resistance [182]. This condition helps us to understand the pathophysiology of metabolic abnormalities associated with insulin resistance. The principal reason for insulin resistance in lipodystrophy is that the excess energy cannot be stored in the AT, which is secondary to the total lack of adipocyte expandability. At this point, the body is unable to store energy in the subcutaneous adipose depots, so it stores fat at ectopic sites in the liver, with a decrease of the adipokines levels, such as leptin and hormones secreted from the AT [183]. Therefore, lipodystrophies may occur from an abnormal preadipocytes development, adipocyte differentiation dysregulation, or increased adipocyte death [184].

The study of lipodystrophy patients provides unique insights into the role of adipose tissue in human health and contributes an important perspective on dysfunctional AT.

## 5. Adipogenesis Modulation

As mentioned before, AT is susceptible of being regulated or modified by different external agents or situations (cold, diet, exercise, drugs, hormones and pollutants), so gene activation and transcription modulation are also crucial in AT storage. An example is the transcription factors, such as Krüppel-like factors (KLFs) and micro ribonucleic acids (miRNAs).

### 5.1. Krüppel-Like Factors

These transcription factors are a family of zinc finger proteins, related to cell proliferation, differentiation, and tissue development [185,186]. Eighteen members have been identified, and Wu and Wang describe very completely, in their review, the adipogenesis-related KLFs. There are five of them involved in the promotion of these processes and three in their inhibition.

The first KLF to be found in the adipogenesis process was KLF15 [187], which is expressed in metabolically active organs, such as the fat, liver, and muscle, suggesting its potential role in metabolism [188,189,190]. KLF15 is induced in response to fasting in both the liver and skeletal muscle tissues and is critical in gluconeogenesis and FA utilization [189,191,192,193]. Additionally, other reports found that KLF15 is highly expressed in AT [188]. Thus, KLF15 may be required in adipocyte biology and be critical for the modulation of adipocyte lipid turnover. AT KLF15 interferes with triglyceride synthesis genes and inhibits lipolysis, provoking lipid storage by insulin [194]. Mori et al. have indicated that KLF15 is a downstream effector of C/EBPβ and δ, as well as PPARγ, to induce white adipocyte differentiation [195]. In some obese mice models, KLF15 expression was lower than in control animals [196], so KLF15 is considered a main component of amino acid, glucose and lipid metabolism in WAT [187].

Besides, KLF5 was identified as a regulator of smooth muscle cells in vascular disease, and also a downstream object of CEBPβ, δ and PPARγ in WAT. Overexpression of these KLFs promotes spontaneously white adipocyte differentiation by up-regulating the transcription factors mentioned above. Recently, a *KLF5* knockout mice strain showed much less WAT mass than the wildtype, indicating that KLF5 also plays a role in WAT development [197].

KLF4 participates in the later stage of adipogenesis [187], and KLF9 has been reported to be required in WAT adipogenesis, but its expression is not sufficient to terminate the adipocyte differentiation process [198].

BAT is also regulated by KLFs, and in this tissue, it is mainly KLF11 and KLF15 that are responsible for BAT differentiation, which activates the UCP1 promoter. However, together or alone, they do not induce adipogenesis, suggesting that they are necessary but not sufficient for the process [199].

In contrast, some KLFs counteract the effect of others regulating and balancing the physiology of adipose tissue. Some examples of this inhibiting function are KLF2, KLF3, and KLF7.

KLF2 was originally involved in lung development; now, it is known to be highly expressed in human preadipocytes and abruptly diminished in adipocyte differentiation. It has a significant down-regulating activity by decreasing the intracellular lipid accumulation [187,200].

KLF3 and 7 have a similar function as KLF2 by repressing transcription [201] and inhibiting preadipocyte differentiation [202].

### 5.2. miRNAs in Adipogenesis

Only 2% of the transcriptional information encodes for proteins, and the remaining genes are in charge of a different class of non-coding RNAs. There are genes associated with non-coding RNAs, which modulate physiological and pathological pathways. miRNAs are an example of non-coding RNAs. miRNAs can act locally to regulate mRNA expression.

Yang et al. found that miRNAs can regulate the gene expression of lipid metabolism by blocking its mRNA translation [203]. Additionally, Thomou et al. demonstrated that circulating miR-containing exosomes impact AT [204].

While miRNA-150 has been found to be expressed in the lymph nodes and spleen and associated with the up-regulation of the development of mature T and B cells, Garvey et al. found that miR-150 targets the mRNA expression of genes, regulating the lipid metabolism and cytokine expression in MФ and WAT and altering the lipid accumulation in adipocytes, including the adiponectin receptor 2 (AdipoR2) [205]. miR-150 may play a role in energy balance and body weight, and another direct target of miR-150 is PGC-1α, which regulates BAT lipid metabolism and obesity-related insulin resistance and inflammation [206]. The brown adipogenesis is regulated in a coordinated way between several transcriptional factors, such as PPARα and γ, CREB, PGC-1α [207], PGC-1β [208], and C/EBPs [209]. PPARγ is for both WAT and BAT development, and its adipogenesis is due to the differences in the expression of PPARγ co-regulators. miR-27 is a central upstream suppressor of the principal brown fat transcriptional regulators, Prdm16, CREB, PPARα, and PGC13, regulating PPARγ directly and PGC-1α indirectly. Thus, miR-27 orchestrates SAT and BAT brown adipogenesis, after cold exposure, in an autonomous manner [210].

Another example is miRNA-374a-5p, which regulates the inflammation process of WAT in obesity [211]. As there are many such examples, and not all of them are going to be discussed in this work, we suggest verifying the reviews by Brandãoa et al., 2017 and Arner et al., 2015 [212,213]. Of all varieties of miRNAs, some can interact with others to equilibrate the physiological functions. miRNAs can also circulate in plasma and are related to the signaling between cells. It has been proposed that these miR (142-3p, 140-5p, 15a, 520c-3p and 423-5p) can be considered candidate as biomarkers for risk estimation and association with morbid obesity [214]. These miRNAs can travel alone or in vesicles. Lately, these vesicles are gaining importance because of their potential as therapeutic targets. Scholars are trying to reestablish cellular communication, using the replacement or injection of extracellular vesicles with miRNAs, to repair severe damages in some lipodystrophies [14].

Novel chemically engineered oligonucleotides, termed ‘antagomirs’, are efficient and specific silencers of endogenous miRNAs. They are powerful tools for silencing specific miRNAs and may be useful therapeutic strategies for silencing them in disease [215].

## 6. Conclusions

In conclusion, AT is a very complex organ that protects against lipotoxicity in response to FFA overload, but overnutrition, promoted by our lifestyle, leads to excessive adiposity. At that moment, AT transitions into a dysfunctional state, where it loses natural control. Adipogenesis, in physiological conditions, is characterized by a pro-inflammatory state, angiogenesis, and the healthy release of adipokines, but it can become dangerous to health if it results in a hypoxic state, causing uncontrolled inflammation and the synthesis and release of harmful free fatty acids. That is why it is crucial to keep this system under control with a healthy diet and exercise. Additionally, it is necessary to continue investigations in order to develop new approaches to treating the associated diseases.

## Figures and Tables

**Table 1:** Human adipose tissue classification, distribution, and function. In this table, the function of human adipose tissue, and how it is classified and distributed, is described. As can be seen, it is very diverse, can be located in all areas of the body and also has a specific function, depending on its location. AT is a vital tissue and is present in almost all parts of the organism. WAT: white adipose tissue; BAT: brown adipose tissue; BMAT: bone marrow adipose tissue; FFA: free fatty acids; SAT: subcutaneous adipose tissue; VAT: visceral adipose tissue; UCP: uncoupling protein.

AT Type	Subdivision	Localization	Function	Reference
BAT		Supraclavicular neck mediastinum	Body thermoregulation ↑ mitochondria ↑ UCP1 energy expenditure	[18,21]
		Paravertebral suprarenal		
BEIGE		SAT to VAT BAT	Colocalized, inducible and transient tissue	[22,23]
WAT	VAT (upper)	perigonadal (pgWAT)	Cushioning thermoregulating energy storage metabolically active secretes adipokines	[18]
		retroperitoneal (rWAT)		
		mesenteric (mWAT)		
		perirenal (prWAT)		
		omental (oWAT)		
		epicardial/pericardial		
	SAT (lower)	Abdominal gluteal femoral	Insulation energy storage adipokines least harmful site of lipid storage	[18]
		deep (dSAT)	More harmful than VAT inflammatory cytokines	[15]
		pink (piSAT) mammary glands	transdifferentiating in mammary glands	[24]
		dermal (dWAT)	wound healing hair follicles thermogenic larger adipocytes and not hematopoietic	[15]
BMAT		Constitutive cBMAT	distal skeleton, spine, and proximal limb bones	[15]
		Regulated rBMAT	Hematopoietic respond to metabolic signals adiponectin source ↑ adipocytes by age and provides FFA to bone	[15]

## Abbreviations

AMP	adenosine monophosphate
AT	adipose tissue
ATGL	adipose triglyceride lipase
ATP	adenosine triphosphate
BAT	brown adipose tissue
BMAL1	circadian gene
BMP	bone morphogenic protein
CC	circadian clock
CD	cluster of differentiation
CLOCK	circadian gene
CRY	crytochrome circadian gene
DAG	diacylglycerol
dSAT	deep subcutaneous adipose tissue
dWAT	dermal adipose tissue
FA	fatty acid
FABP	fatty-Acid-binding Protein
FAS	fatty acid synthase
FFA	free fatty acids
G3P	glycerol-3-phosphate
Glut	glucose transporter
HDACs	histone deacetylases
HIF-1α	hypoxia-inducible factor 1 alpha
HK	hexokinase
HSL	hormone-sensitive lipase
IL	interleukin
iNOS	inducible nitric oxide synthase
KLF	Krüppel-like factor
LPL	lipoprotein lipase
MФ	macrophages
MAT	bone marrow adipose tissue
MCP-1	monocyte chemotactic protein-1
MetS	metabolic syndrome
miRNAs	micro ribonucleic acids
NOX	NADPH oxidase
PGC-1α	peroxisome proliferated receptor γ coactivator 1 α
PKA	protein kinase A
Plins	perpilins
PPAR	peroxisome proliferator-activated receptor
ROS	reactive oxygen species
TLR	toll-like receptors
Treg	regulatory T cells
SAT	subcutaneous adipose tissue
SREBP	sterol regulatory element-binding protein
TG	triglycerides
TNF	tumour necrosis factor
UCP	uncoupling protein
VAT	visceral adipose tissue
VEGF	vascular endothelial growth factor
WAT	white adipose tissue
